# Involvement of miR-106b in tumorigenic actions of both prolactin and estradiol

**DOI:** 10.18632/oncotarget.16755

**Published:** 2017-03-31

**Authors:** Kuan-Hui Ethan Chen, Karissa Bustamante, Vi Nguyen, Ameae M. Walker

**Affiliations:** ^1^ Division of Biomedical Sciences, University of California, Riverside, CA 92521, USA

**Keywords:** miR-106b, prolactin, estradiol, p21, breast and prostate cancer

## Abstract

Prolactin promotes a variety of cancers by an array of different mechanisms. Here, we have investigated prolactin's inhibitory effect on expression of the cell cycle-regulating protein, p21. Using a miRNA array, we identified a number of miRNAs upregulated by prolactin treatment, but one in particular that was strongly induced by prolactin and predicted to bind to the 3′UTR of p21 mRNA, miR-106b. By creating a p21 mRNA 3′UTR-luciferase mRNA construct, we demonstrated degradation of the construct in response to prolactin in human breast, prostate and ovarian cancer cell lines. Increased expression of miR-106b replicated, and anti-miR-106b counteracted, the effects of prolactin on degradation of the 3′UTR construct, p21 mRNA levels, and cell proliferation in breast (T47D) and prostate (PC3) cancer cells. Increased expression of miR-106b also stimulated migration of the very epithelioid T47D cell line. By contrast, anti-miR-106b dramatically decreased expression of the mesenchymal markers, *SNAIL-2, TWIST-2, VIMENTIN, and FIBRONECTIN*. Using signaling pathway inhibitors and the 3′UTR construct, induction of miR-106b by prolactin was determined to be mediated through the MAPK/ERK and PI3K/Akt pathways and not through Jak2/Stat5 in both T47D and PC3 cells. Prolactin activation of MAPK/ERK and PI3K/Akt also activates ERα in the absence of an ERα ligand. 17β-estradiol promoted degradation of the construct in both cell lines and pre-incubation in the estrogen antagonist, Fulvestrant, blocked the ability of both prolactin and 17β-estradiol to induce the construct-degrading activity. Together, these data support a convergence of the prolactin and 17β-estradiol miR-106b-elevating signaling pathways at ERα.

## INTRODUCTION

Prolactin is a peptide hormone produced and released from many tissues, but in largest quantity from the pituitary gland [1]. The best known functions of prolactin are in the mammary gland where prolactin promotes gland development during pregnancy, the synthesis of milk, and maintenance of milk secretion [2]. However, prolactin also functions in immune regulation [3–5], adipocyte differentiation [6, 7], islet proliferation [8, 9] and regulation of bone density [10, 11]. In addition, more and more evidence has accumulated indicating that prolactin promotes tumorigenesis in various types of cancers, including breast [12–14], prostate [14, 15], ovary [14, 16], liver [17], and brain [18], as well as stimulating the growth of pituitary tumors [19, 20]. One of the tumor-promoting mechanisms is decreased expression of the cell cycle inhibitor CDKN1A, also known as p21 [14]. A reduction in p21 expression may have a direct effect on cell proliferation, but may also contribute to anti-estrogen and other drug resistance in some cancers [21–26]. As might be expected, the expression of p21 is tightly regulated by several molecules, including p53 [27], BRCA1 [14, 28], TGF-β [29], RAR/RXR [30] and the vitamin D receptor [31]. In addition to expression of p21, cellular localization of p21 is also related to the therapeutic resistance [32]. Restriction of p21 to the cytosol is regulated through post-translational modification on threonine 145, which is phosphorylated by Akt, PKA or pim-1 kinase [32–34]. Therefore, regulation of p21 expression and localization is of importance in both cancer development and therapeutic resistance.

Estrogens also contribute to progression of a number of cancers [35–38]. We and others have identified a synergy between prolactin and estrogen in the promotion of breast cancer [39–42], and the ability of prolactin to activate the estrogen receptor α (ERα) in the absence of an ERα ligand [40, 41]. Thus, studying the interplay between these two hormones in cancer progression is important.

We have previously demonstrated that prolactin regulates p21 transcription through inhibition of BRCA1 function [14]. In the current study, we have determined the existence of an additional mechanism through which prolactin decreases the amount of p21, the induction of miR-106b, a mechanism duplicated by estradiol. Further, that increased expression of miR-106b causes increased cell number and enhanced migratory capacity.

## RESULTS

### Prolactin effects on miRNA expression

**Figure 1:**
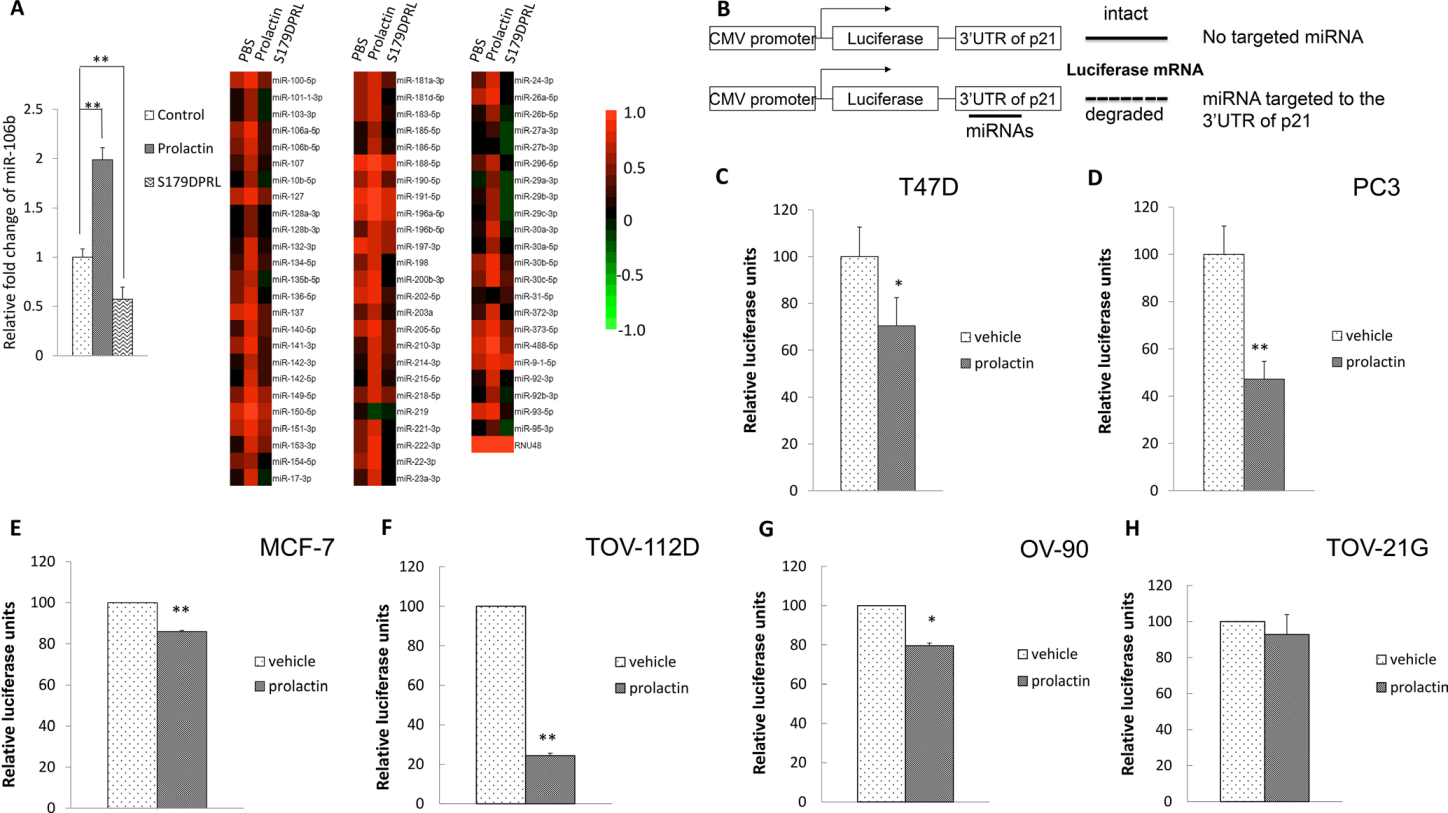
Prolactin treatment induced miR-106b targeted to the 3′UTR of the p21 in cancers (**A**) T47D cells were treated with vehicle control, 100 ng/mL prolactin or 100 ng/mL selective prolactin receptor modulator, S179DPRL, for 72 hours. Total RNA was extracted and 5 μg total RNA from each treatment was subjected to miRNA array. (**B**) Illustration of luciferase-3′UTR assay to detect miRNA production. When there was miRNA production that bound to the 3′UTR region, this would lead to degradation of luciferase mRNA and reduced luciferase signals. The breast cancer cell line, T47D (**C**) and prostate cancer cell line, PC3 cells (**D**) were transfected with luciferase-p21 mRNA 3′UTR plasmid followed by 100 ng/mL prolactin treatment for 72 hours. This analysis was also performed in one additional breast cancer cell line, MCF7 (**E**) and 3 ovarian cancer cell lines, TOV-112D (**F**), OV-90 (**G**) and TOV-21G (**H**) with prolactin treatment limited to a 24 hour period in these cases. Luciferase activity was then measured. Each luciferase activity value from vehicle-treated cells was set as 100. Data are presented as the mean ± S.D.; *P < 0.05 and **P < 0.01 (compared with vehicle treatment).

To explore how prolactin might reduce expression of p21 mRNA, we performed a miRNA microarray in T-47D breast cancer cells. To help refine our analysis, we used both prolactin, which reduces, and the selective prolactin receptor modulator, S179DPRL, which elevates, p21 [14, 43]. Prolactin and S179DPRL bind to the same receptors [44], and have some functions in common [45], but in regard to cell proliferation, prolactin stimulates [46], while S179DPRL inhibits (14, 43, 45, 46). Using a 2-fold change in expression as our stringency cutoff, there were 21 miRNAs upregulated by prolactin (Table 1). Among these miRNAs, miR-106b has been shown to target to the 3′ UTR of p21 mRNA directly [47]. None of the other 20 miRNAs upregulated by prolactin is predicted to interact directly with any region of p21 mRNA using online database miRDB analysis [48, 49]. Most these other miRNAs have been reported to be associated with reduced expression of p21 indirectly [50–52]. miR-107, miR-153 and miR-142-3p indirectly reduce p21 expression through targeting the upstream regulators, FOXO1, PTEN and FOXO4, respectively [50–52]. We therefore focused further analysis on miR-106b. Consistent with a p21-elevating effect of S179DPRL [46], S179DPRL also decreased expression of miR106b by 43% compared to the PBS control (Figure 1A).

**Table 1:** Upregulated miRNAs (> 2 fold) by prolactin

miRNA	Fold
miR-10b-5p	2.648065866
miR-95-3p	2.365516801
miR-92-3p	2.278490033
miR-92b-3p	2.26453033
miR-27a-3p	2.250477655
miR-26b-5p	2.225025659
miR-17-3p	2.210714719
miR-215-5p	2.196169084
miR-101-1-3p	2.173145188
miR-30a-3p	2.164483133
miR-142-5p	2.154083454
miR-214-3p	2.127021904
miR-132-3p	2.100969166
miR-142-3p	2.097205532
miR-185-5p	2.090877882
miR-153-3p	2.047451196
miR-372-3p	2.029475511
miR-186-5p	2.011486344
miR-103-3p	2.006442321
miR-107	2.000847941
miR-106b-5p	2.000066166

To substantiate a role for miRNA in the degradation of p21 mRNA in the current study, we created a plasmid construct by conjugating the 3′UTR of p21 mRNA downstream of a luciferase reporter. If there were miRNAs targeted to the 3′UTR, this would lead to degradation of luciferase mRNA and therefore would reduce translated luciferase activity (Figure 1B). Consistent with the microarray experiment, when T-47D cells were transfected with this plasmid and treated with prolactin or vehicle for 72 hours, treatment with prolactin significantly reduced the luciferase activity (Figure 1C). In addition to T-47D cells, this effect was also reproduced in human prostate cancer PC3 cells (Figure 1D). To further illustrate this was not limited to the 2 cell types we focused on for most of the study, we also examined a second human breast cancer cell line, MCF-7, and 3 human ovarian cancer cell lines. For these additional cell lines the effect of prolactin on luciferase activity is shown at 24 hours in Figure 1E–1H. Prolactin caused a significant reduction in luciferase activity in MCF-7, TOV-112D, and OV 90 cells in this time frame, indicating prolactin induction of miRNA targeted to the 3′UTR of p21 mRNA. The degree of response was very cell line dependent, ranging from a 15% reduction of luciferase activity in MCF7 cells to an 80% reduction in TOV-112D cells.

**Figure 2:**
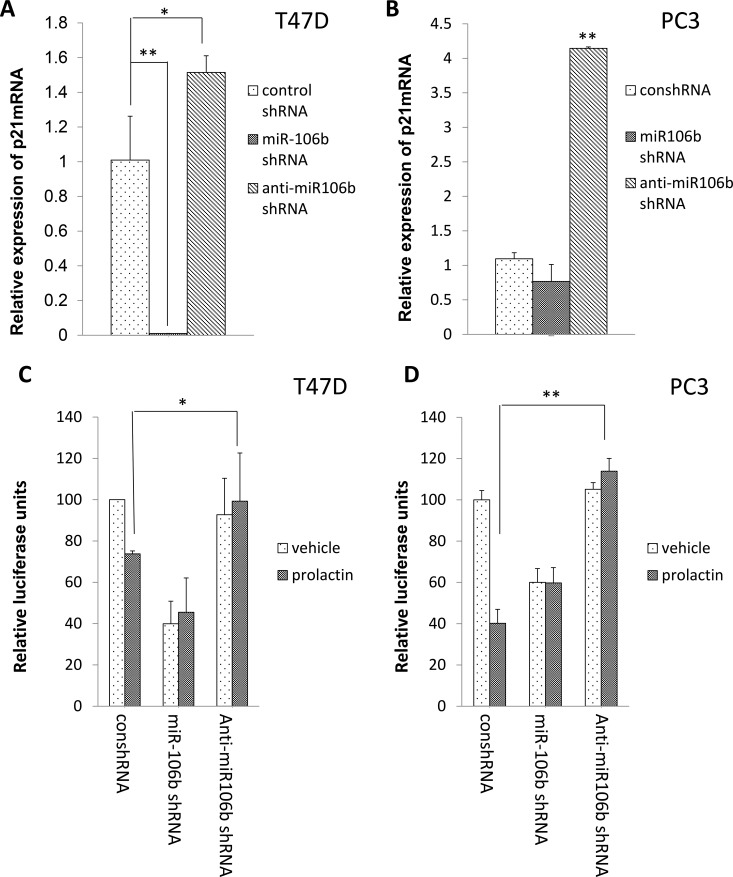
Inhibition of miR-106b by anti-miR106b shRNA blocked prolactin mediated effect on both the 3′UTR of p21 mRNA and p21 mRNA expression Increased expression of miR-106b shRNA reduced p21 mRNA in T47D cells (**A**) while increased expression of anti-miR-106b shRNA resulted in more p21 mRNA in PC3 cells (**B**). The expression of p21 transcript was normalized to GAPDH. Each transcript level from non-treated cells was set as 1. Both T47D (**C**) and PC3 (**D**) cells were co-transfected with luciferase-p21 3′UTR plasmid and control shRNA/ miR-106b shRNA/ anti-miR-106b shRNA and treated with 100 ng/mL prolactin or vehicle for 72 hours. Luciferase activity was then measured. Each luciferase activity value from vehicle treated cells was set as 100. Data are presented as mean ± S.D.; *P < 0.05 and **P < 0.01 (compared with vehicle treatment).

To demonstrate that the effect of prolactin was mediated through miR-106b, we constructed shRNA plasmids to be used to increase expression of either miR-106b or anti-miR-106b. Increased expression of miR-106b essentially eliminated p21 mRNA in T47D cells, whereas anti-miR-106b increased p21 mRNA. In PC3 cells, which grow more rapidly and are less epithelioid, increased expression of miR106b had no effect, while increased expression of anti-miR106b quadrupled the expression of p21 mRNA (Figure 2A and 2B). Using the luciferase assay to assess the effect of increased expression with and without prolactin, increased expression of miR-106b reduced luciferase activity and prolactin treatment did not augment this effect. However, the luciferase-lowering effect of prolactin was blocked when there was increased expression of anti-miR-106b (Figure 2C and 2D). Collectively, these results show that prolactin induced the production of miR-106b and that this then targeted the 3′ UTR of p21 mRNA.

### Cells expressing more miR-106b were more aggressive

**Figure 3:**
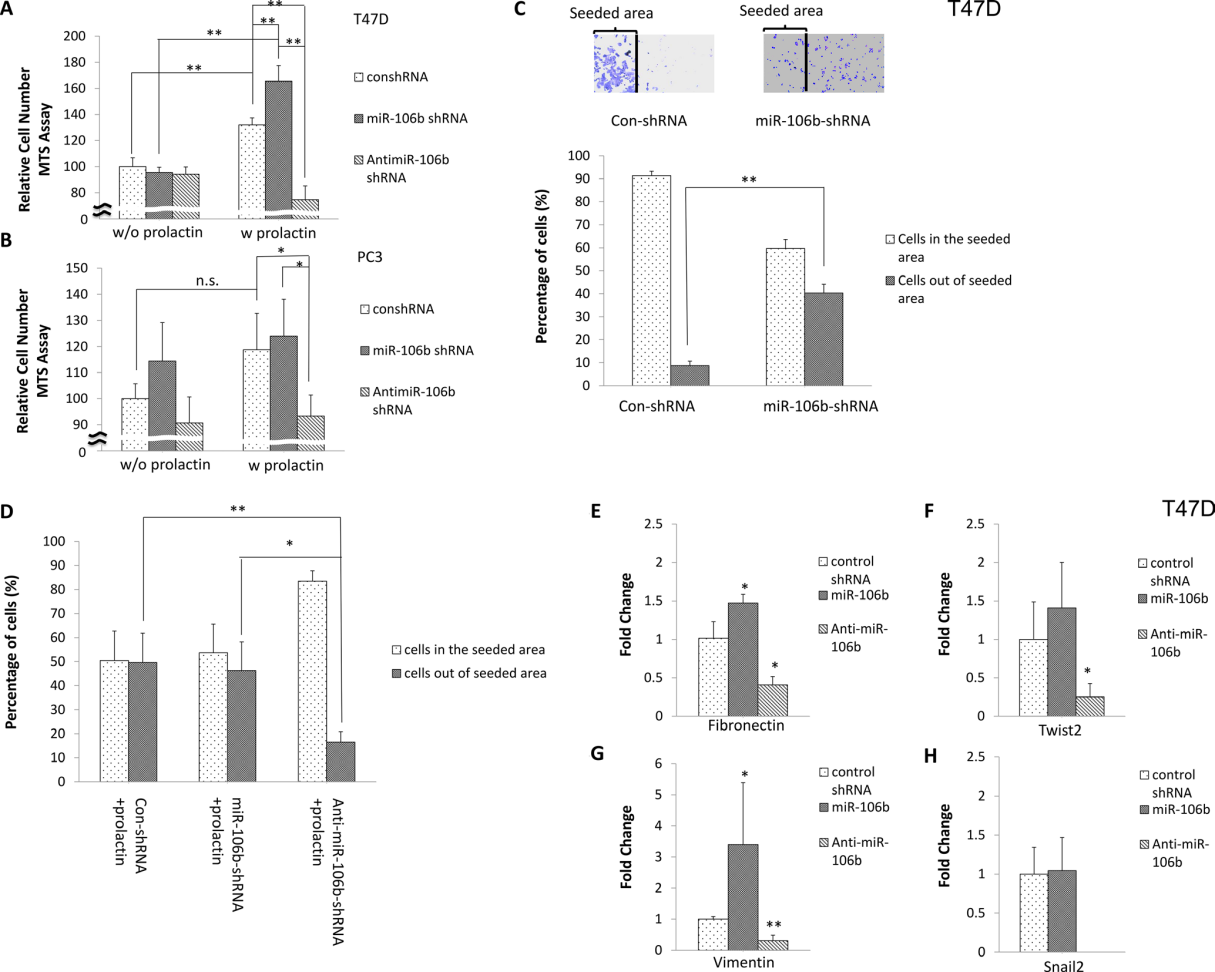
Effect of miR-106b on cell number, cell migration and mesenchymal gene expression 10,000 T47D (**A**) and PC3 (**B**) cells were transfected with control shRNA, miR-106b shRNA or anti-miR-106b shRNA plasmid and treated with vehicle (left panel) or 100 ng/mL prolactin (right panel) for 72 hours. Relative cell number was then measured by MTS assay. Data are presented as the mean ± S.D.; *P < 0.05. T47D cells were seeded in the constrained area and transfected with control shRNA or miR-106b shRNA plasmid in the absence (**C**) or presence (**D**) of prolactin. After 72 hours, the distribution of cells was counted in both the original seeded area and outside this area. (**E**–**H**). T47D cells were transfected with control shRNA, miR-106b shRNA or anti-miR-106b shRNA plasmid in the presence of 100 ng/mL prolactin for 72 hours. The expression of mesenchymal genes was analyzed by real time PCR. The expression of transcript was normalized to GAPDH. Each transcript level from control shRNA transfected cells was set as 1. Data are presented as mean ± S.D.; *P < 0.05 and **P < 0.01 (compared with control shRNA transfection).

To determine the outcome of upregulation of miR-106b in cancer, we first examined effects on relative cell number, as assessed by MTS assay, with increased expression of miR-106b or anti-miR106b in the absence or presence of prolactin. As seen in Figure 3A, increased expression of miR-106b or anti-miR106b in T47D cells did not cause any effect on cell number in the absence of prolactin. Prolactin alone (with control shRNA) increased the relative number of cells by 35% during the 72-hour incubation. Additional expression of miR-106b in the presence of prolactin doubled the response. By contrast, increased expression of anti-miR-106b in the presence of prolactin decreased cell number below that without prolactin. In the PC3 cell line, which grows rapidly, increased expression of miR-106b or incubation with prolactin did not significantly increase cell number. However, increased expression of anti-miR-106b significantly inhibited growth of cells in the presence of prolactin (Figure 3B). These results suggest that increased levels of miR-106b alone are insufficient to cause an increase in cell number, but that by reducing p21 levels, any promotion of proliferation can be enhanced. By contrast, increased levels of anti-miR-106b effectively block proliferation.

We next examined whether increased expression of miR-106b affected cell migration. T-47D cells were chosen for this analysis because they are very epithelioid in nature and migration rates are normally very slow. Cells were seeded in a constrained area in the center of the well and then transfected with control shRNA or miR-106b shRNA plasmid. As seen in Figure 3C, most control shRNA-transfected cells remained in the original area. However, cells with increased expression of miR-106b dispersed to a greater radius. Incubation in prolactin (with control shRNA), had a similar effect to transfection with miR-106b shRNA (compare Figure 3C and 3D). However, combining increased expression of miR-106b with incubation in prolactin did not further increase the amount of migration. As would be predicted from these results, expression of anti-miR-106b decreased migration to about the level seen in the absence of prolactin and additional miR-106b, thereby confirming a role for miR-106b in the promotion of migration.

To further confirm an effect on migratory capacity, we examined the expression of genes characteristic of an epithelial to mesenchymal transition. Increased expression of miR-106b with prolactin treatment in T47D cells increased expression of fibronectin and vimentin, whereas increased expression of anti-miR-106b along with prolactin treatment decreased the expression of fibronectin, vimentin, twist2 and snail2, with the last reduced to undetectable levels, leaving us no ability to determine significance levels (Figure 3E–3H). Taken together, these results suggest a high impact of miR-106b on proliferative and metastatic aspects of tumor progression.

### The induction of miRNAs by prolactin was mediated through MAPK, PI3K and activation of the estrogen receptor

**Figure 4:**
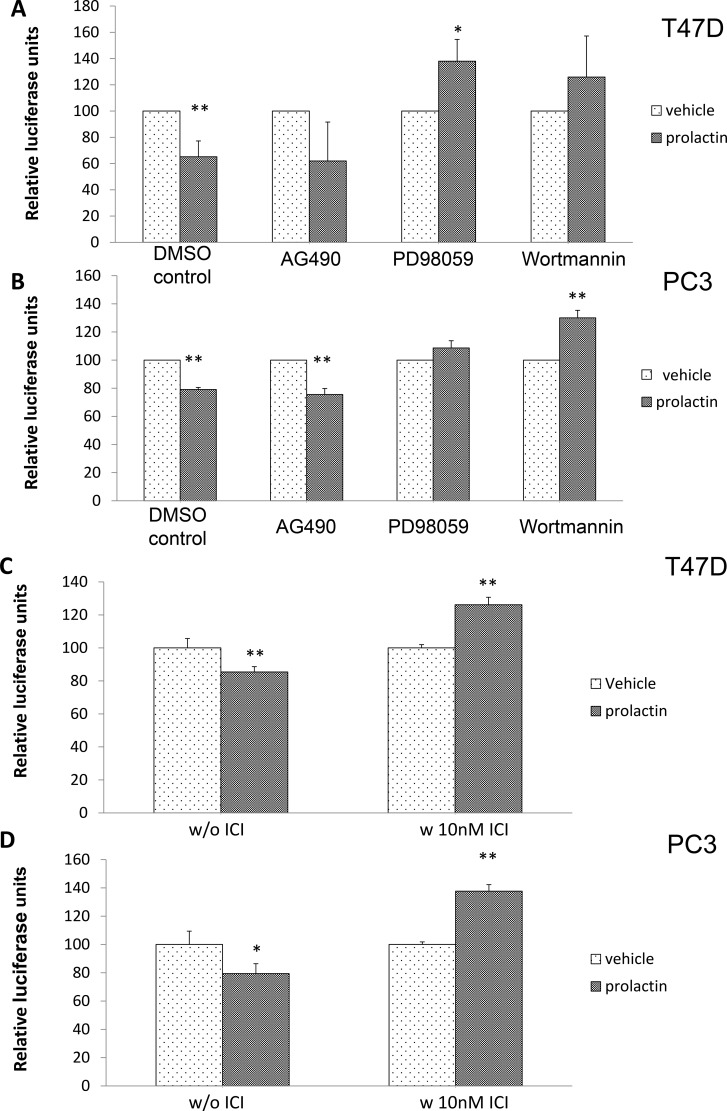
Prolactin activation of MAPK/ERK, PI3/Akt and ERα are involved in miRNA induction targeted to the 3′UTR of p21 mRNA T47D (**A**) and PC3 cells (**B**) were transfected with the luciferase-p21 3′UTR plasmid and pre-treated with DMSO (control), 10 μM AG490, 20 μM PD98059 or 1 μM Wortmannin for 4 hours prior to the addition of 100 ng/mL prolactin or vehicle for 20 hours. Luciferase activity was then measured. Both T47D (**C**) and PC3 (**D**) cells were transfected with the luciferase-p21 3′UTR plasmid and treated with DMSO control (left panel) or 10 nM ICI-182780 (right panel) in the presence of 100 ng/mL prolactin for 20 hours. Luciferase activity was then measured. Each luciferase activity value from vehicle-treated cells was set as 100. Data are presented as mean ± S.D.; *P < 0.05 and **P < 0.01 (compared with vehicle treatment).

To identify which prolactin signaling pathway/s contributed to the induction of the miRNA(s), PC3 and T-47D cells were pre-treated with different signaling inhibitors (PD98059 for MAPK/ERK, Wortmannin for PI3K/Akt, AG490 for Jak2/Stat5) and transfected with the luciferase-p21 3′ UTR plasmid. The effect of these inhibitors was also validated by examining activation of the appropriate signaling molecules using Western blot (data not shown). Because these inhibitors negatively impact cell viability when used for extended periods, effects on luciferase activity were measured 24 hours after transfection and incubation with the inhibitors, with 20 of those hours in the absence or presence of prolactin. This protocol was sufficient for all inhibitors except wortmannin, which still had a small effect (~20% in both the absence and presence of prolactin) on cell viability in the 24 hour time frame (data not shown). To compensate, data are expressed as a percent of luciferase activity in the absence of prolactin. i.e. the graph is specifically showing the effect of the inhibitors on the induction of p21-targeting miRNA as a function of prolactin treatment. Given that we are looking at increases in luciferase activity if the signaling pathway is blocked, an increase in translated and active luciferase is unlikely to be the result of a decrease in cell viability. While one tends to think of the Jak2/Stat5 pathway as being of primary importance to prolactin signaling, AG490 had no effect on the induction of miRNA by prolactin. However, prolactin failed to reduce luciferase activity when PD98059 or wortmannin was present in the medium (Figure 4A in T47D cells and 4B in PC3 cells). Thus, prolactin signals through MAPK/ERK and PI3K/Akt to elevate miRNA targeting the p21 mRNA 3′UTR. Wortmannin is a more potent PI3K/Akt inhibitor than the commonly used LY294002. However, at higher concentrations, wortmannin may also inhibit other PI3 related kinases such as mTOR, DNA-PK, and ATM [53]. As the concentration we used may also block these other kinases, we therefore cannot exclude the possibility that these other kinases might also be involved in the miRNA production.

Prolactin signaling through MAPK/ERK and PI3K/Akt pathways also activates ERα in the absence of an ERα ligand [40]. We therefore examined the potential role for activated ERα in the production of miR-106b. Cells were pre-treated with the ERα inhibitor, ICI-182780, and then transfected with the luciferase-p21 3′UTR plasmid. As seen in Figure 4C and 4D, addition of ICI-182780 not only blocked the luciferase degradation promoted by prolactin, but also increased the amount of luciferase activity compared to control. In other words, blockade/degradation of ERα inhibited the ability of prolactin to elevate the miRNA in both breast cancer and prostate cancer cell lines.

**Figure 5:**
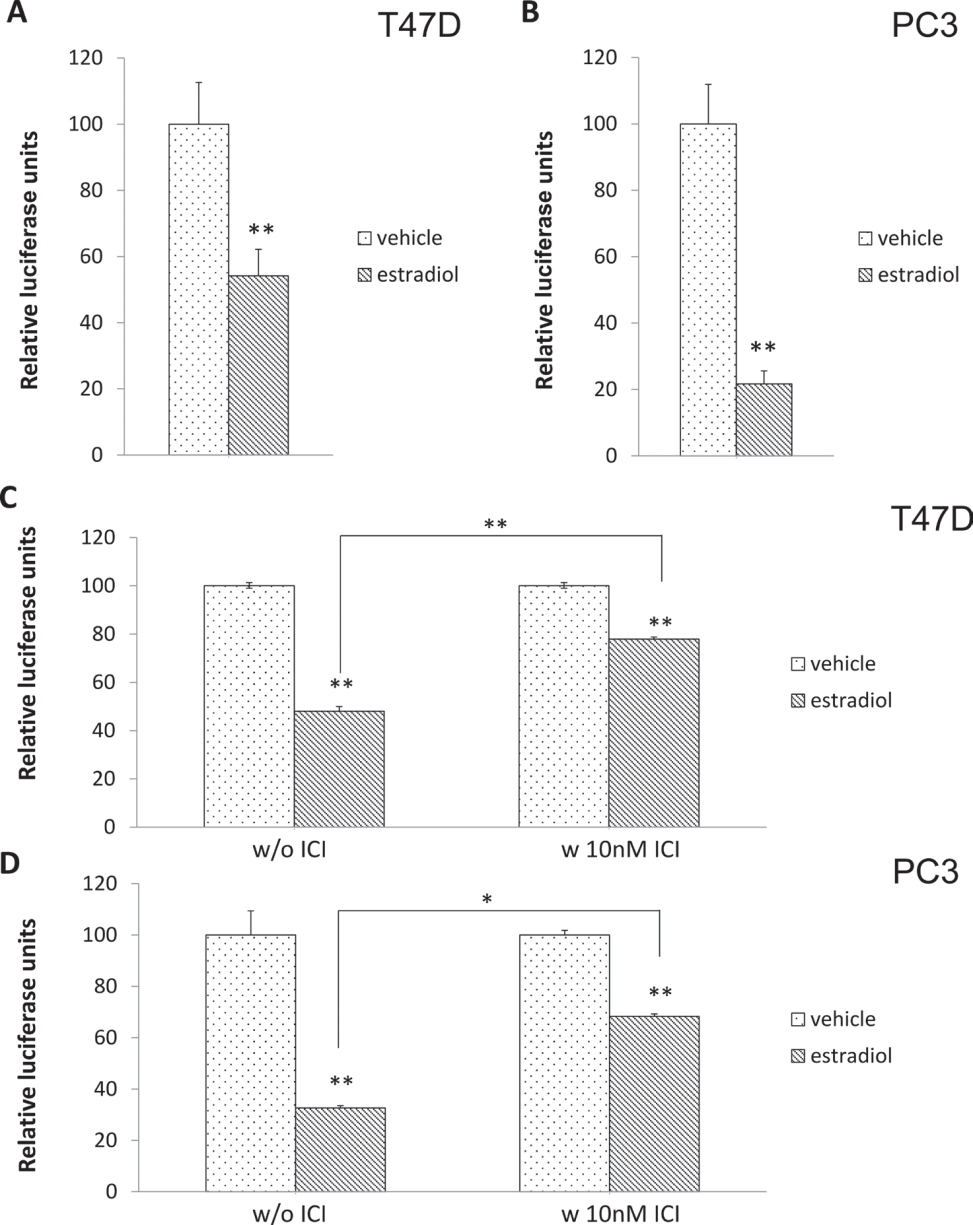
Activation of ERα by 17β-estradiol significantly induced miRNA production that bound to the 3′UTR of the p21 mRNA construct T47D (**A**) and PC3 cells (**B**) were transfected with the luciferase-p21 3′UTR plasmid followed by 1 nM 17β-estradiol treatment. Luciferase activity was then measured after 72 hours. Both T47D (**C**) and PC3 (**D**) cells were transfected with the luciferase-p21 3′UTR plasmid and treated with DMSO control (left panel) or 10 nM ICI-182780 (right panel) in the presence of 1 nM 17β-estradiol for 20 hours. Luciferase activity was then measured. Each luciferase activity value from vehicle-treated cells was set as 100. Data are presented as mean ± S.D.; *P < 0.05 and **P < 0.01 (compared with vehicle treatment).

To further demonstrate that activation of ERα can result in the production of miRNA targeting the p21 mRNA 3′UTR, cells were transfected with the luciferase construct and treated with 17β-estradiol. As seen in Figure 5A and 5B, estradiol induces miRNAs targeting the 3′UTR of p21 mRNA in both breast cancer and prostate cancer cell lines. In addition, when cells were pretreated with ICI-182780, the reduction of luciferase activity by estradiol was relieved (Figure 5C and 5D).

Taken together, these data indicate that activated ERα mediates the effect of both prolactin and estradiol on the production of miRNA targeting the 3′UTR of the p21 mRNA.

## DISCUSSION

There are many publications supporting roles for prolactin in the progression of a variety of cancers [13, 16, 54–56]. These include various pro-proliferative, anti-apoptotic and pro-metastatic roles. In addition, prolactin may contribute to therapeutic drug resistance [57, 58]. The development of resistance to therapy can occur by a variety of mechanisms, but loss of p21 expression is a common factor [21, 23–26]. Identification of mechanisms governing p21 expression may lead to development of new therapeutics, as well as to strategies to prevent development of resistance to current therapies.

To explore how prolactin might reduce expression of p21 mRNA, we performed a miRNA microarray in T-47D breast cancer cells. Using a 2-fold change as the stringency cutoff, 21 miRNAs were upregulated by prolactin. To help sort through which of these to focus on, we used two approaches. The first approach was to compare with the effect of S179DPRL. While prolactin decreases expression of p21, S179DPRL increases p21 [14, 43]. Although the mechanism for increasing p21 might not have been through decreased production of miRNAs, we could at least expect to be able to eliminate some miRNAs from further consideration if they were also increased by S179DPRL. miR-9-1-5p is a good example of one eliminated. The second approach was to examine these 21 miRNAs for predicted direct interactions with any region of p21 mRNA by online database miRDB search [48, 49]. miR-106b was the only predicted miRNA and had been shown to interact with p21 mRNA directly [47], although its role as a mediator of any of prolactin's functions had not been described. For the remaining 20 miRNAs, literature suggested an indirect regulation on p21 expression. For example, miR-107, miR-153, and miR-142-3p had been shown to reduce p21 expression through targeting the upstream regulators, FOXO1, PTEN and FOXO4, respectively [50–52]. Our focus was then on miR-106b. miR-106b was not only increased by prolactin, but decreased by S179DPRL, suggesting in fact that at least part of the mechanism used by S179DPRL to elevate p21 is a reduction in miR-106b.

Using the artificial construct, the data presented show that prolactin stimulates the production of a miRNA that targets the 3′ UTR of p21 mRNA and that this is duplicated by increased expression of miR-106b and antagonized by anti-miR-106b. Furthermore, that the effects of miR-106b and anti-miR106b in the luciferase assay are translated to effects on levels of p21 mRNA. In T47D cells, increased expression of miR-106b essentially eliminated p21 mRNA, while anti-miR-106b had little effect. By contrast, miR-106b had little effect on p21 mRNA in PC3 cells, while anti-miR-106b quadrupled the amount of p21 mRNA. These results are consistent with different baseline levels of p21 in the two cell lines, with levels of p21 higher in the slower growing and more epithelioid T47D cell line. However, the amount of p21 mRNA did not translate to a change in cell number unless prolactin was also present. Thus, prolactin has additional functions that affect cell number beyond those mediated by miR-106b. To this point, in a previous publication [14] we demonstrated that prolactin regulates transcription of the p21 gene by interfering with BRCA1 transactivation. Therefore, in response to prolactin, one might expect a greater reduction in p21 and a greater effect on cell number than that brought about only by increased expression of miR-106b. Alternatively, a greater effect of prolactin on cell number versus that produced by increased expression of miR-106b might be expected as a result of prolactin's ability to stimulate the cell cycle [59, 60]. Given the ability of increased miR-106b to reduce p21 mRNA to essentially zero (albeit mRNA and not protein) and that this does not cause an increase in cell number, the most likely of these two explanations is that the additional effect of prolactin is pro-proliferative rather than anti-apoptotic.

In addition to oncogenic miR-106b, prolactin also upregulated other oncomirs such as members of the miR-17/92 family. Interestingly, the only 2 miRNAs downregulated by prolactin, miR-219 and miR-31, exhibit antitumor effects [61–63]. These data are supportive of multiple tumorigenic roles for prolactin.

In addition to miR-106b, miR-106a was also induced by prolactin (1.5 fold), but to a level below the stringency of our microarray analysis. This miRNA is known to directly target the 3′UTR of p21. Therefore, the effect of prolactin to decrease p21 mRNA could be regulated by both miR-106b and miR-106a, but, based on relative expression levels, the contribution from miR-106a is less.

The data also demonstrate that increased expression of miR-106b contributes to cell migration, which is consistent with previous studies by others. For example, Gong et al. [64] and Yau et al. [65] showed increased metastasis and migration in breast and hepatocellular carcinoma cells, respectively, with increased expression of miR-106b. Furthermore, induction of miR-106b is found in multiple types of cancers, including breast, prostate and ovarian cancers [66–69]. Prolactin also increases metastatic spread [55, 56], and the current experiments suggest that some of this may be through induction of miR-106b, although this was not directly addressed. Rather, we showed that prolactin induced miR106b, and miR-106b in turn increased expression of fibronectin and vimentin, while anti-miR-106b decreased expression of fibronectin, vimentin, twist2 and snail2. As p21 has been demonstrated to be an important inhibitor of the epithelial-to-mesenchymal transition [70, 71], our data are consistent with the literature.

There are other reported targets for miR-106b, such as PTEN, SMAD7 and REST [72–75] and downregulation of these targets by miR-106b also contributes to tumor progression. For instance, a decreased PTEN level would also cause reduced p21, and decreased SMAD-7 would lead to increased epithelial to mesenchymal transition [72, 75]. Thus, there are likely multiple integrated effects.

When looking at an effect of prolactin, one might expect it to be related to the level of expression of prolactin receptors. We did see a wide range of responsiveness to prolactin in the luciferase assay and to some extent there is a correlation. For example, in the three ovarian cancer cell lines, expression of total prolactin receptor is high in TOV-112D, modest in OV-90 and low in the TOV-21G [16]. This pattern is consistent with the miRNA production by prolactin stimulation in these three cell lines. In addition, T-47D cells express more prolactin receptors than MCF-7 cells [76] and T47D cells showed a greater response than MCF7 cells. However, PC3 cells have few prolactin receptors and yet showed a substantial response to prolactin in the luciferase assay. There must therefore be other factors at play governing this particular response to prolactin. One possible factor may be the status of p53. Under most conditions, p21 expression is p53 dependent [27]. Consistent with this suggestion, the cell lines with wild type p53, MCF-7 and TOV-21G cells, have lower induction of miR-106b by prolactin, while cell lines with mutant p53, TOV-112D, OV-90 and T47D, or with null p53, PC3, showed greater induction of miR-106b by prolactin.

We have previously shown that prolactin activation of Stat5 causes it to form a complex with BRCA1 that prevents BRCA1 from transactivating the p21 promoter [14]. We therefore expected the Jak2/Stat5 signaling pathway to be involved in the production of miR-106b since both actions lead to reduced p21. However, based on the use of signaling pathway inhibitors, Jak2 is not involved. Rather, signaling from the prolactin receptor to increased expression of miR-106b is through MAPK/ERK and PI3K/Akt. Thus, multiple signaling pathways regulate p21 levels. These pathways are also activated by membrane ERα [77]. We therefore considered the possibility that there may be crosstalk and possible synergies between estradiol and prolactin signaling in the production of miR-106b, especially since there is synergy in terms of cell proliferation [40]. However, even though both estradiol and prolactin had similar effects in the luciferase assay, blockade/degradation of ERα blocked both the prolactin and estradiol effects, thereby demonstrating that ERα is situated between prolactin signaling and the production of miR-106b. How then does prolactin elevate expression of miR-106b? Interestingly, we have also previously shown that prolactin activation of the MAPK/ERK and PI3K/Akt pathways leads to serine-118 phosphorylation of ERα and its activation in the absence of an ERα ligand [40]. Thus, even when the use of aromatase inhibitors deprives a patient of estradiol, prolactin would still be capable of reducing expression of p21 and promoting tumor progression, although prolactin expression would also usually be reduced with aromatase inhibitors [78, 79]. However, other positive influences on prolactin production such as stress [80] and the use of some anti-psychotics [81, 82] would still be operative and capable of influencing tumor promotion through this mechanism.

**Figure 6:**
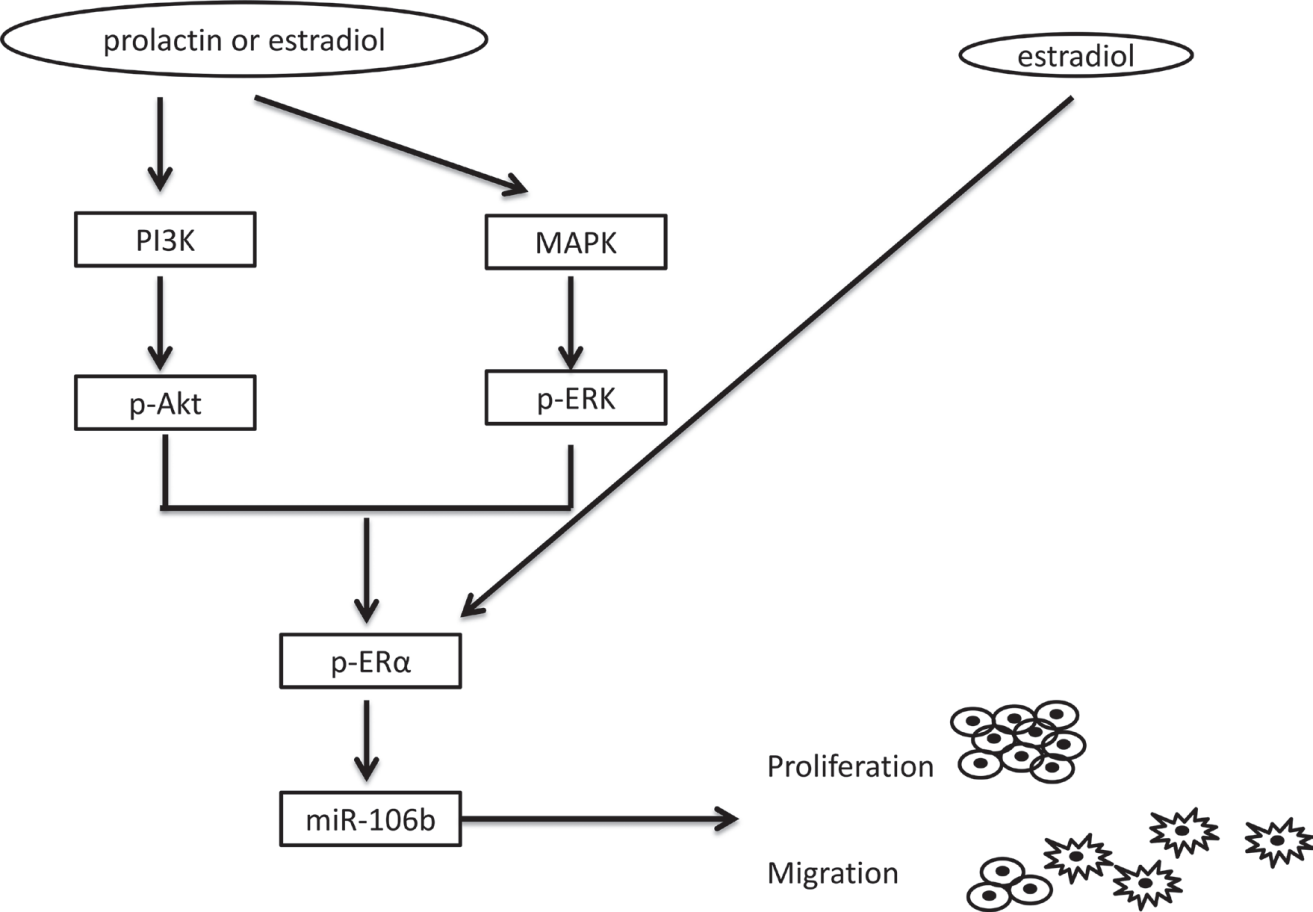
Model of the deduced pathway leading to tumor progression Prolactin or estradiol activate Akt and MAPK signaling pathways via their membrane receptors. This leads to activation of ERα. Alternatively or additionally, ERα may be activated directly through the classical intracellular pathway. Once ERα is activated by one or both hormones, expression of miR-106b is increased. Increased expression of miR-106b facilitates cell proliferation through a decrease in p21 and increases cell migration through upregulation of messenchymal gene expression.

In the current study, we have determined that upregulation of miR-106b by prolactin likely contributes to tumor malignancy. This upregulation is mediated through the Akt and MAPK pathways and the downstream activation of ERα. This is diagrammed in Figure 6. Thus, miR-106b or its regulated events might serve as therapeutic targets for both breast and prostate cancers.

## MATERIALS AND METHODS

### Cell culture

All cell lines (human breast cancer cell lines, T47D and MCF-7, the human prostate cancer cell line, PC3, and human ovarian cancer cell lines, TOV-112D, OV-90 and TOV-21G) were freshly purchased from ATCC (Manassas, USA) where they were authenticated utilizing Short Tandem Repeat analysis. Cells were routinely cultured in RPMI 1640 medium supplemented with 10% fetal bovine serum (FBS) and were used for experiments with passage numbers < 20.

### Production of reporter construct and pMIR luciferase assay

The 3′UTR fragment of p21 was first amplified using the primer pair in Table 2. The amplified fragment was digested with MluI /HindIII and then ligated to the pMIR reporter vector with the same restriction enzyme digestion (Applied Biosystems, Waltham, USA) using T4 DNA ligase (New England Biolabs, Ipswich, USA). Two hundred and fifty thousand cancer cells were seeded in each well of a 12-well plate and co-transfected with the luciferase-p21 3′UTR plasmid and a β-galactosidase plasmid in the absence (Dulbecco's phosphate buffered saline – DPBS) or presence of 100 ng/mL prolactin or 1nM 17β-estradiol for 72 hours. After 72 hours, cells were washed 2 times with DPBS and then lyzed in luciferase lysis buffer (Promega, Madison, USA). Following transfer of the lysate to a 96-well plate, the luciferin substrate (Promega, Madison, USA) was added. When examining the effect of signaling inhibitors, a shorter incubation period of 24 hours was utilized since prolonged use of signaling inhibitors is toxic to cells. For these assays, 20,000 cancer cells were seeded per well in a 96-well plate and co-transfected with the construct and β-galactosidase plasmids in the presence of the inhibitors such as to incubate cells in 20 μM PD98059 (EMD Biosciences, La Jolla, USA), 1 μM wortmannin (EMD Biosciences, La Jolla, USA), 10 μM ICI-178280 (also known as Fulvestrant) (Sigma-Aldrich, St. Louis, USA) or 10 μM AG490 (EMD Biosciences, La Jolla, USA). Four hours after transfection, medium was changed to fresh medium with the same concentrations of inhibitors in the absence or presence of prolactin (100 ng/mL). Luciferase activity was measured 24 hours after initiation of transfection, but 20 hours after the addition of prolactin. Transfection efficiency was normalized to β-galactosidase expression. Control experiments using the standard in the kit demonstrated that none of the inhibitors directly affected luciferase activity.

**Table 2:** Primers used for cloning or PCR

**miR-106b shRNA**	F: CTTCCTGTCATAAAGTGCTGACAGTGCAGATCTGCAGTCTGGAGTTTCA R: TGACAGGAAGTAAAGTGCTGACAGTGCAGATCGAGATCTTGGGCCTCT
**p21 3′UTR**	F: CGACGCGTCCGCCCACAGGAAG R: CCAAGCTTGAGCACCTGCTGTA
**p21**	F: CGACTGTGATGCGCTAATGG R: GGCGTTTGGAGTGGTAGAAATC
**Vimentin**	F: GGACCAGCTAACCAACGACA R: AAGGTCAAGACGTGCCAGAG
**Fibronectin**	F: ACAAGCATGTCTCTCTGCCA R: TTTGCATCTTGGTTGGCTGC
**Snail2**	F: CAACGCCTCCAAAAAGCCAA R: ACTCACTCGCCCCAAAGATG
**Twist2**	F: CAGAGCGACGAGATGGACAA R: TGCATCCCAATTCCACTTGC
**GAPDH**	F: CCTCCTGTTCGACAGTCAGC R: TGGAATTTGCCATGGGTGGA

### shRNA plasmid construction

The construction of shRNA was performed as described by [29], but in the suresilencing shRNA plasmid (SABioscience, Frederick, USA). The primers used for miR-106b shRNA construction are listed in Table 2. The outcome of increased expression of miR-106b was measured by p21 mRNA expression using real time PCR (primers in Table 2) and by the luciferase-p21 3′UTR assay described in 4.2.

### Cell migration and epithelial-to-mesenchymal transition (EMT)

One hundred thousand cancer cells, transfected with control shRNA or miR-106b shRNA were placed in a constrained area around the center of well. After 72 hours of incubation, cells were fixed with methanol for 5 min, followed by staining with 0.03% methylene blue for 10 min. Cell movement was analyzed by comparing cell distribution between control shRNA- and shRNA-miR106-treated cells. EMT status was examined by the expression of the mesenchymal genes, snail2, fibronectin, twist2 and vimentin using real-time PCR. Gene expression was normalized to the housekeeping genes, GAPDH and β-actin. All primer information is listed in Table 2.

### MTS relative cell number assay

Ten thousand cancer cells were seeded in each well of a 96-well plate and transfected with miR-106b shRNA or control plasmid. Relative cell number was assessed using a mitochondrial tetrazolium reduction assay (MTS, Promega, Madison, USA) at 72 hours.

### miRNA array

Human T47D cells were seeded in 35mm wells in the presence of vehicle, 100 ng/mL prolactin or S179DPRL for 72 hours. Cells were then collected for miRNA expression analysis following the instructions in the Signosis Human microRNA Array II (AP-0002) (Signosis, Santa Clara, USA).

### Statistical analyses

All experiments were conducted a minimum of 3 times using a minimum of triplicates on each occasion. Statistical significance was determined by ANOVA with post tests and Bonferroni corrections for multiple comparisons, where applicable. A *P value* < 0.05 was considered significant.

## Abbreviations

None.
